# Comparison of Blue®m and chlorhexidine mouthwash on oral health of intubated patients in intensive care unit

**DOI:** 10.1590/0103-644020256537

**Published:** 2025-12-08

**Authors:** Tereza C. Teixeira, Renan A.G. Ribeiro, Valeriana Mascella, Tatiana M. Deliberador

**Affiliations:** 1 Department HCM, Santa Casa de Misericórdia de Sorocaba, Sorocaba, Brazil; 2 Department of Implantology, Latin American Institute of Odontological Research and Education - ILAPEO College, Curitiba, Brazil

**Keywords:** oral hygiene, intensive care units, mouthwashes, chlorhexidine, blue®m

## Abstract

This research aims to evaluate whether blue®m mouthwash can be used as a suitable alternative to chlorhexidine mouthwash for improving the oral health of intubated patients in ICUs. This is a randomized clinical study. The sample comprised 50 mechanically ventilated adults randomly allocated in a block of 10 to the 0.12% chlorhexidine digluconate (CHX) and the blue®m group. The patients' oral and tracheal secretions were collected in four different periods: T1 - 24h after intubation (without sanitation); T2 - 24h after intubation (with sanitation); T3 - 48h after intubation (without sanitation); T4 - 48h after intubation (with sanitation). Bacterial and fungal identification was performed. The presence of bacteria was graded as abundant, moderate, and scarce. Descriptive and inferential statistics were performed. Fifty patients with a mean age of 62.7 ± 13.3 years (20 to 86 years) were treated, 24 in the CHX group and 26 in the blue®m group. The most frequent pathogens in the patient's oral environment were *Acinetobacter baumanii, Pseudomonas aeruginosa,* and *Klebsiella pneumonia*. Analysis of the effectiveness of both antiseptics indicated statistically significant differences in the median values of the microbial population between collection periods in the CHX (χ2=13.2, p=0.004) and blue®m (χ2=9.49, p=0.023) group. No significant statistical difference in microbial population was found between the groups (T1- p=0.375; T2 - p=0.955; T3 - p=0.765; T4 - p=0.898). Blue®m mouthwash effectively reduces the oral microbial population and does not differ from chlorhexidine in terms of reduction. Blue®m mouthwash can be used as an alternative to chlorhexidine for mechanical ventilator patients in the ICU.

## Resumo

Esta pesquisa tem como objetivo avaliar se o enxaguatório bucal blue®m pode ser usado como uma alternativa adequada ao enxaguatório bucal de clorexidina para melhorar a saúde bucal de pacientes intubados em UTIs. A amostra foi composta por 50 adultos ventilados mecanicamente, alocados aleatoriamente em um bloco de 10 no grupo digluconato de clorexidina 0,12% (CHX) e o grupo blue®m. As secreções orais e traqueais dos pacientes foram coletadas em quatro períodos diferentes: T1 - 24h após a intubação (sem higienização); T2 - 24h após a intubação (com higienização); T3 - 48h após a intubação (sem higienização); T4 - 48h após a intubação (com higienização). Foi realizada a identificação bacteriana e fúngica. A presença de bactérias foi classificada como abundante, moderada e escassa. Estatísticas descritivas e inferenciais foram realizadas. Cinquenta pacientes com idade média de 62,7 ± 13,3 anos (20 a 86 anos) foram tratados, 24 no grupo CHX e 26 no grupo blue®m. Os patógenos mais frequentes no ambiente oral do paciente foram *Acinetobacter baumanii, Pseudomonas aeruginosa* e *Klebsiella pneumonia*. A análise da eficácia de ambos os antissépticos indicou diferenças estatisticamente significativas nos valores medianos da população microbiana entre os períodos de coleta no grupo CHX (χ2=13,2, p=0,004) e blue®m (χ2=9,49, p=0,023). Não foi encontrada diferença estatisticamente significativa na população microbiana entre os grupos (T1- p=0,375; T2 - p=0,955; T3 - p=0,765; T4 - p=0,898). O enxaguante bucal blue®m reduz efetivamente a população microbiana oral e não difere da clorexidina em termos de redução. O enxaguante bucal blue®m pode ser usado como uma alternativa à clorexidina para pacientes de ventilador mecânico na UTI.

**Figure:**
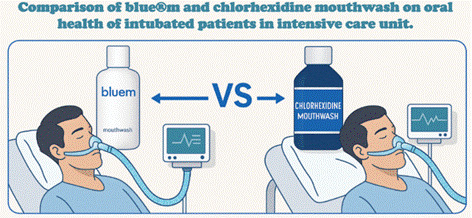

## Introduction

Oral care is essential for patients in the ICU and contributes to general health, especially for those intubated. Maintaining good oral hygiene in these patients prevents oral and dental infections, ventilator-associated pneumonia (VAP), gingivitis, oral bleeding, bad breath, and pain^1^. Due to the high employment of mechanical ventilation in the ICU, VAP is one of the most common nosocomial infections, with an incidence of 23.8% to 36.0%^2^. The aspiration of oral microbial colonies, often attributed to poor oral hygiene, contributes to VAP^3^.

In addition to pharmaceutical strategies, oral hygiene contributes to preventing VAP. A systematic review evaluated the influence of oral hygiene on the reduction of VAP, finding a reduction when different approaches were used, such as antiseptic or toothbrushing ^4^. Over the years, chlorhexidine has been the standard of oral hygiene care in ICU patients, and it has been indicated to decrease the risk of VAP^5^. However, recent meta-analyses suggested that chlorhexidine may not decrease ventilator-associated pneumonia risk in non-cardiac surgery patients, and there is a possibility that chlorhexidine is associated with increased mortality^6^. Adverse events in the oral mucosa were also observed when chlorhexidine was used^7^. This way, evaluating another alternative is essential to continuously improving ICU patients’ health care.

Another alternative product used in oral hygiene that reduces biofilm/plaque formation is blue®m active oxygen. Santos et al. (2023)^8^ evaluated the antimicrobial capacity of blue®m mouthwash against the bacterium *Streptococcus mutans* and its influence on the expression of the gbpA gene, which is related to biofilm formation and bacterial virulence, as well as the cytotoxic effect of blue®m on fibroblast cells. Blue®m mouthwash showed antimicrobial activity and exhibited low cytotoxicity levels, being considered non-toxic at the concentrations tested. The authors suggested the product has therapeutic potential as an alternative agent for controlling oral biofilm.

Studies comparing blue®m and chlorhexidine mouthwash effectiveness and safety in chronic periodontitis, chronic gingivitis, dental implant treatment, and ultrasonic scaling procedures were performed (9-13); however, to the author’s knowledge, this study is the first in hospital dentistry comparing both mouthwashes. Therefore, this research aimed to evaluate whether blue®m mouthwash is effective in reducing the microbial oral population and can be used as a suitable alternative to chlorhexidine mouthwash for improving the oral health of intubated patients in ICUs.

## Materials and methods

### Design

This study was a controlled, randomized clinical trial with two groups. The principal researcher (who performed the intervention), patients (due to their reduced level of consciousness), and the evaluator were unaware of the study groups. The principal researcher received the mouthwash already prepared according to the allocation. The evaluator received the patient sample without knowledge of which mouthwash each group received.

There was no patient or public involvement in the design, conduct, and reporting of the trial. The study protocol was submitted and approved by the Ethics Committee (Sorocaba, Brazil; opinion n°. 001/2024). The investigation was conducted according to the revised principles of the Helsinki Declaration and reported according to CONSORT recommendations. The privacy rights of human subjects were observed, and written informed consent was obtained from each enrolled patient representative. No changes were performed after protocol approval by the Ethics Committee.

This study was designed as an equivalence trial to compare the effects of blue®m and Chlorhexidine mouthwash on the oral health of mechanically ventilated patients in the ICU. The hypothesis is that blue®m mouthwash is not inferior to Chlorhexidine mouthwash.

### Sampling

This study involved the participation of intubated patients admitted to the Hospital Irmandade Santa Casa de Misericórdia de Sorocaba in Sorocaba, located in Brazil, from December 2024 to January 2025.

The sample was selected prospectively and consisted of patients over 18, both sexes, who were admitted to the ICU in mechanical ventilation and whose informed consent was signed by close relatives, such as mother, father, or children. Patients were excluded if they did not meet the inclusion criteria, pregnant women, patients whose informed consent form was not signed by close relatives such as spouse, mother, father, or children, and who died during the data collection period, as well as patients with an indication for extubation.

No previous sample size calculation was performed, since there was no published study using Bluem mouthwash in a hospital context to use as a reference for sample calculation.

### Recruitment and allocation

Patients who met the inclusion criteria were selected, sequentially numbered, and randomly allocated in blocks of 10. They were assigned to groups: Control (0.12% Chlorhexidine digluconate group) and Test (blue®m group). The randomization and allocation were centralized in a third-party person who was responsible for handling the randomization and allocation process. This third-party person was responsible for generating the randomization list using the Excel random function. This list was kept exclusively with this person, who had no role in patient recruitment, clinical assessments, or data analysis.

At the time of each patient’s inclusion, the independent third party was contacted, and then assigned the participant to the respective group. To ensure allocation concealment, the independent third party prepared the intervention materials according to the assigned group and delivered them in a coded form to the investigator. This process guaranteed that the investigators administering the intervention remained blinded to treatment allocation throughout the study.

**Figure 1:**
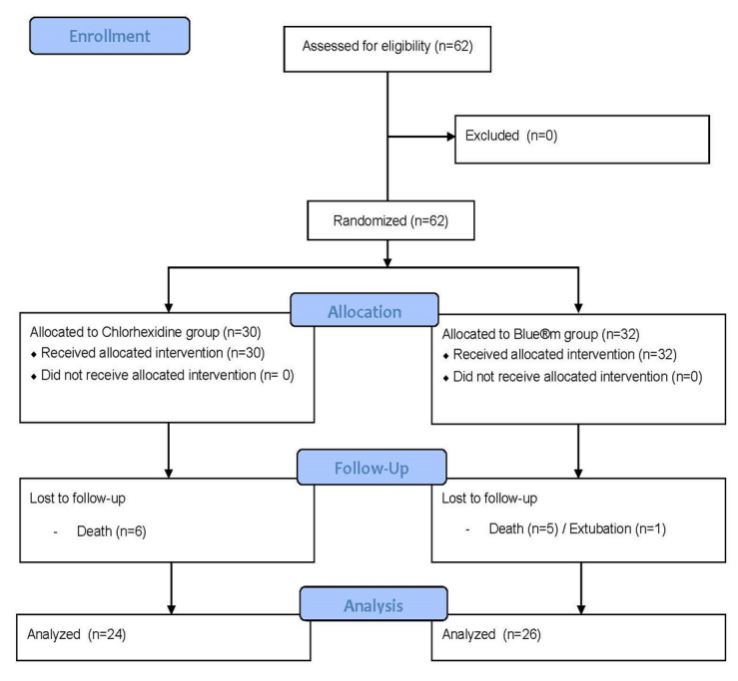
Flow chart of the study design, enrollment, allocation, randomization, follow-up, and analysis of study patients.

Thirty patients were included in the chlorhexidine group and 32 in the blue®m group. However, six patients from each group were excluded due to death and extubation; thus, 50 patients completed the study (Figure 1).

### Procedures

In the Control Group, a 0.12% Chlorhexidine digluconate mouthwash (Indahex, Dores do Indaiá, Brazil) was employed. In contrast, the Test Group used blue®m mouthwash solution (blue®m, Zwolle, Netherlands). A pharmacist added Chlorhexidine digluconate to a container labeled A, and the mouthwash blue®m was added to another container labeled B and was given to the principal researcher. Until the end of the study, only the pharmacist knew about their contents.

In both groups, the cuff pressure was adjusted to between 25 and 20 mmHg before performing oral care using a special manometer to ensure its appropriateness. The headboard was raised to 30 degrees, and the patient’s head was turned to one side. Oral hygiene was performed by aspirating secretions from the oropharynx and applying the solution from the hygiene kit using a swab to all teeth, tongue, and the surface of the oral mucosa. The research team, composed of dental surgeons, performed the entire process.

Personal protective equipment was used to collect tracheal secretions: a mask and protective eye goggles, hand sanitation, and sterile gloves. A sterile 12-gauge suction probe was inserted and connected to the sterile collector, and the secretion was collected (a minimum volume of 5 ml) (if the volume was insufficient due to the accumulation of secretions, 0.9% saline solution was added). The research team underwent training to calibrate the collection.

### Outcome

The patients' oral and tracheal secretion (buccal mucosa) (obtained from the tube material) was collected as bacterial culture material. The material was collected using a sterile swab (for collection and transport). The material collected from both groups was identified with the patient code and stored in a Stuart transport medium (4 ml). All collected material samples were sent to the hospital's analysis laboratories within a maximum interval of 4 hours, where microbiological tests were performed.

Bacterial and fungi identification was performed after culturing the bacteria in the samples by staining and corresponding biochemical methods to verify the presence of the primary pathogens associated with infections resulting from orotracheal intubation and VAP. The presence of bacteria was graduated in abundant, moderate, and scarce at each collection time.

The collections followed the scheme below:

- T1: 24h after intubation (without sanitation).
- T2: 24h after intubation (with sanitation).
- T3: 48h after intubation (without sanitation).
- T4: 48h after intubation (with sanitation).

### Data analysis

The data were analyzed using Jamovi software version 2.6.19. Quantitative variables were described by mean, standard deviation, minimum, and maximum. For qualitative variables, absolute and relative frequencies were provided. Before performing the inferential statistics, the microbial population result was transformed to ordinal data where “abundant” corresponded to 3, “moderate” to 2, and “scarce” to 1. A Friedman test, followed by a post-hoc Durbin-Conover test, was performed to evaluate the effectiveness of both groups in reducing the microbial population. The Mann-Whitney test was used to compare the microbial population between the two groups. The per-protocol analysis was used. The significance level was set at less than 0.05. 

## Results

Sixty-two patients were enrolled in the study between December 2024 and January 2025. However, twelve patients were excluded due to death and extubation, resulting in fifty patients treated with a mean age of 62.7 ± 13.3 years (varying from 20 to 86 years). The principal investigator delivered the comparator and the intervention to all patients. Patients were hospitalized for different reasons. The most frequent in the blue®m group was heart failure (3; 11.54%), pneumonia (3; 11.54%), stroke (2; 7.69%), and cardiac arrest (2; 7.69%). In the Chlorhexidine group, the most frequent reason for hospitalization was stroke (4; 16.67%) and lung disease (2; 8.33%). Depending on the clinical conditions, each patient received a different concomitant care in accordance with their needs.

The main bacterial pathogens in the patient's oral environment were *Acinetobacter baumanii, Pseudomonas aeruginosa,* and *Klebsiella pneumonia.* However, *Enterobacter hormaechei, Acinetobacter ursingii, Serratia marcescens, Enterobacter cloacae, Morganella,organii, Raoultella ornithinolytica, Klebsiella aerogenes, Staphylococcus haemolyticus, Escherichia coli, Stenotrophomonas maltophilia, Corynebacterium striatum, Citrobacter koseri, Proteus mirabilis, Citrobacter freundii, and Staphylococcus epidermidis* were also found. *Candida albicans* and *Candida tropicalis* were the predominant fungi found.

The Friedman test results were significant, based on an alpha value of 0.05, χ^2^=9.49, p=0.023, indicating statistically significant differences in the median values of the microbial population between collection periods in the blue®m group. Pairwise comparisons were examined between each combination of variables. The results of the multiple comparisons indicated significant differences, based on an alpha value of 0.05, between the following variable pairs: T1-T2, T2-T3, and T3-T4. Table 1 presents the results of the post-hoc analysis.

**Table 1:** Evaluation of the difference in microbial population at each collection period per group.

Group	Time	Median (1^st^ - 3^rd^ Quartiles)	p-value
Blue®m	T1-T2	3.00 (3.00 - 3.00) - 3.00 (2.00 - 3.00)	0.023^*^
	T1-T3	3.00 (3.00 - 3.00) - 3.00 (3.00 - 3.00)	0.732
	T1-T4	3.00 (3.00 - 3.00) - 3.00 (2.00 - 3.00)	0.075
	T2-T3	3.00 (2.00 - 3.00) - 3.00 (3.00 - 3.00)	0.009^*^
	T2-T4	3.00 (2.00 - 3.00) - 3.00 (2.00 - 3.00)	0.607
	T3-T4	3.00 (3.00 - 3.00) - 3.00 (2.00 - 3.00)	0.035^*^
Chlorhexidine	T1-T2	3.00 (3.00 - 3.00) - 3.00 (2.00 - 3.00)	0.002^*^
	T1-T3	3.00 (3.00 - 3.00) - 3.00 (3.00 - 3.00)	0.695
	T1-T4	3.00 (3.00 - 3.00) - 3.00 (2.00 - 3.00)	0.008^*^
	T2-T3	3.00 (2.00 - 3.00) - 3.00 (3.00 - 3.00)	0.008^*^
	T2-T4	3.00 (2.00 - 3.00) - 3.00 (2.00 - 3.00)	0.695
	T3-T4	3.00 (3.00 - 3.00) - 3.00 (2.00 - 3.00)	0.021^*^

*Statistical difference p<0.05. Friedman and Durbin-Conover Test, p<0.05.

The Friedman test results for the chlorhexidine group were significant, based on an alpha value of 0.05, χ^2^=13.2, p=0.004, indicating significant differences in the median values of the microbial population between collection periods. The multiple comparisons results showed significant differences, based on an alpha value of 0.05, between the following variable pairs: T1-T2, T1-T4, T2-T3, and T3-T4. Table 1 presents the results of the post-hoc analysis.

**Figure 2:**
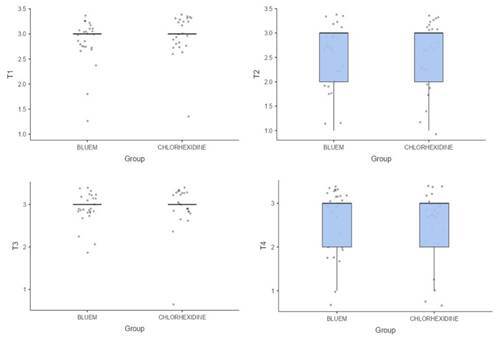
Distribution of microbial presence at each time.

The comparison of the microbial oral population between both groups at each time point is described in Table 2. The two-tailed Mann-Whitney U test result was not significant based on an alpha value of 0.05 in all collection periods (T1 - p=0.375; T2 - p=0.955; T3 - p=0.765; T4 - p=0.898). The microbial oral population was similar between groups, with both T1 and T3 groups presenting a high population, whereas T2 and T4 groups had a lower population. Figure 2 presents a box plot of the ranks of microbial populations by group and collection period. 

**Table 2:** Comparison of microbial population between groups at each time point.

Time	Group	Median (1^st^ - 3^rd^ Quartiles)	p-value
T1	Blue®m	3 (3 - 3)	0.375
	Chlorhexidine	3 (3 - 3)	
T2	Blue®m	3 (2 - 3)	0.955
	Chlorhexidine	3 (2 - 3)	
T3	Blue®m	3 (3 - 3)	0.765
	Chlorhexidine	3 (3 - 3)	
T4	Blue®m	3 (2 - 3)	0.898
	Chlorhexidine	3 (2 - 3)	

Mann-Whitney Test, p<0.05.

## Discussion

This study aimed to determine the effectiveness of blue®m mouthwash in reducing the oral microbial population in ICU patients and confirm its non-inferiority to Chlorhexidine mouthwash. The results showed that the oral microbial population decreased in both groups, and there was no difference regarding this population in all collection periods between the groups, proving the blue®m mouthwash non-inferiority.

Since direct aspiration is the primary responsibility for VAP, oral decontamination is indicated. Over the years, studies have demonstrated that applying topical oral antibiotics decreases oropharynx contamination and helps prevent VAP. However, a concern regarding the development of multi-resistant bacteria has been raised. Thus, studies have been designed to analyze the use of antiseptics in oral hygiene to prevent VAP instead of antibiotics. Due to chlorhexidine's extensive history of safety and efficacy, it was the first antiseptic choice^14^.

Similar to the results found in our study, scientific literature has already demonstrated chlorhexidine's effectiveness in reducing the microbial population and directly decreasing the occurrence of VAP in ICU patients^15^. However, besides chlorhexidine's history of use, it was associated with mucosa irritation, erosive oral lesions, ulcerations, white/yellow plaques, and bleeding mucosa^7^. Additionally, studies associate chlorhexidine with higher mortality risks due to aspiration of this substance and the development of acute respiratory distress syndrome^16^. A review suggested an increase in isolates presenting a certain tolerance to chlorhexidine, which could have terrible consequences, such as an increase in hospitalization time^17^. Thus, the indiscriminate use of chlorhexidine in oral care may be harmful.

Given this evidence, alternative antiseptics have been brought to the forefront. blue®m has been emerging as a safety alternative for different clinical targets. This product is an active oxygen therapy. Active oxygen has already been reported to be beneficial to wound healing of oral tissues due to its influence on angiogenesis^18^. Indeed, a case series of 14 patients analyzed the impact of blue®m in the healing of pre-prosthetic surgery to remove hyperplasia of the maxillary posterior and anterior ridge. The authors find that Bluem reduced the pain and signs of inflammation^19^.

Some studies have already compared the effectiveness of blue®m and chlorhexidine mouthwashes. Studies compared the use of blue®m and chlorhexidine mouthwashes in the nonsurgical management of moderately deep pockets in chronic periodontitis. The blue®m showed a statistically significant reduction in clinical and microbiological parameters associated with soft tissue health^10^. Another study evaluated the efficacy of both mouthwashes and their effects on plaque, calculus, and gingival inflammation in patients with generalized chronic gingivitis. Both were effective, and no statistical differences were found. The authors suggested that blue®m can be a safe alternative to chlorhexidine ^11^.

Pawane et al. evaluated the antibacterial efficacy of blue®m in patients who have undergone dental implant treatment and compared it with chlorhexidine. They found a reduction in total bacterial copy count in both groups from baseline, indicating that blue®m can be a safe alternative to chlorhexidine in reducing the microbial load post-operatively^12^. Sindhusha et al. evaluated the bacterial load in the aerosols emitted during the ultrasonic scaling procedure and found a statistically significant reduction in the blue®m group compared to chlorhexidine ^13^.

Studies observed that blue®m is antimicrobial against *S. mutans*, promoting bactericidal, bacteriostatic, and antibiofilm effects at low concentrations^8^. The effectiveness of blue®m gel in periodontal disease was analyzed. The gel decreased the microbial population, periodontal inflammation, and bleeding on probing^20^. Besides all these blue®m studies, to the authors' knowledge, this is the first study to analyze the efficacy of blue®m mouthwash in reducing the oral microbial population in ICU patients and comparing it with chlorhexidine in this hospitalized population.

No adverse event was observed in this study. Cytotoxicity tests were performed to confirm blue®m^’^s safety, and minimal or no cytotoxic effect was observed. Additionally, no oral tissue damage was observed^8^ ^,^ ^21^ ^,^ ^22^. All these published studies and our results suggest that blue®m is safe and can be used as an oral antiseptic to reduce the oral microbial population.

Various studies evaluated the bacterial and fungi pathogens of VAP. The most frequent pathogens found were *Acinetobacter baumannii, Pseudomonas aeruginosa, Stenotrophomonas maltophilia, Klebsiella pneumoniae, and Serratia marcescens.* Other pathogens were found in addition, such as *Enterobacteriaceae*, *Klebsiella* spp., *Escherichia coli*, *Acinetobacter* spp., *Enterobacter* spp., *Staphylococcus epidermidis, Staphylococcus haemolyticus, Candida albicans, and Candida tropicalis* ^23^ ^,^ ^24^. Similarly, these pathogens were also present in our sample.

Finally, studies have reported the influence of oral hygiene on reducing the risk of VAP in ICU patients^25^. However, oral care must be performed by trained and specialized healthcare professionals to avoid the aspiration of contaminated fluids into the respiratory tract^4^. A dental surgeon or technician in the ICU is fundamental to an efficient and safe oral hygiene procedure in a sensitive patient like the ICU patient.

The most significant limitation of this study is its small sample size (24 in the Chlorhexidine group and 26 in the blue®m group). This study also has limitations concerning infants and children. Additionally, due to the short-term analysis, the long-term effect of blue®m was not evaluated. To complement this study's results, future studies should examine the impact of blue®m mouthwash on the incidence of VAP and long-term adverse effects.

This clinical study has not been previously registered on a public clinical trials registry platform, which constitutes a relevant methodological limitation. Despite this limitation, the study was conducted according to a protocol previously defined, planned, and approved by the Ethics Committee, which detailed the inclusion criteria, participant allocation, collection times, and outcomes. Additionally, data were collected prospectively and analyzed according to the planned design, with standardized inclusion and exclusion criteria consistently applied to all participants. Such measures aim to minimize the risk of selection bias and ensure the reproducibility of the findings. The protocol and data can be accessed upon request to the author.

## Conclusion

According to the present study, blue®m mouthwash effectively reduces the oral microbial population and does not differ from chlorhexidine in terms of reduction. Blue®m mouthwash can be used as an alternative to chlorhexidine for mechanical ventilator patients in the ICU.
